# Author Correction: Dynamic reconfiguration of functional brain networks during working memory training

**DOI:** 10.1038/s41467-020-17747-8

**Published:** 2020-07-30

**Authors:** Karolina Finc, Kamil Bonna, Xiaosong He, David M. Lydon-Staley, Simone Kühn, Włodzisław Duch, Danielle S. Bassett

**Affiliations:** 1 Centre for Modern Interdisciplinary Technologies, Nicolaus Copernicus University, Toruń, Poland; 2 Department of Informatics, Faculty of Physics, Astronomy and Informatics, Nicolaus Copernicus University, Toruń, Poland; 3 Department of Bioengineering, School of Engineering and Applied Science, University of Pennsylvania, Philadelphia, PA USA; 4 Annenberg School for Communication, University of Pennsylvania, Philadelphia, PA USA; 5 Lise Meitner Group for Environmental Neuroscience, Max Planck Institute for Human Development, Berlin, Germany; 6 University Medical Center Hamburg–Eppendorf, Hamburg, Germany; 7 Department of Electrical & Systems Engineering, School of Engineering and Applied Science, University of Pennsylvania, Philadelphia, PA USA; 8 Department of Neurology, Perelman School of Medicine, University of Pennsylvania, Philadelphia, PA USA; 9 Department of Physics & Astronomy, School of Arts and Sciences, University of Pennsylvania, Philadelphia, PA USA; 10 Department of Psychiatry, Perelman School of Medicine, University of Pennsylvania, Philadelphia, PA USA; 11 Santa Fe Institute, Santa Fe, NM 87501 USA

**Keywords:** Working memory, Human behaviour

Correction to: *Nature Communications* 10.1038/s41467-020-15631-z, published online 15 May 2020.

The original version of this Article omitted the following from the Acknowledgements:

Publication fees were funded by the Polish National Agency for Academic Exchange under a project name Open NCU.

This has now been corrected in both the PDF and HTML versions of the Article.

