# Supplementary material for: Dynamic reconfiguration of functional brain networks during working memory training
Source: Nat Commun. 2020 May 15;11:2435. doi: 10.1038/s41467-020-15631-z (PMC7229188; doi:10.1038/s41467-020-15631-z)
Supplement: Supplementary file 1 — Supplementary Information [file 41467_2020_15631_MOESM1_ESM.pdf]

Dynamic reconfiguration of functional brain networks during working memory training

Finc *et al.*

SUPPLEMENTARY INFORMATION

Contents

1 Supplementary Figures **2**

2 Supplementary Tables **23**

3 Supplementary Methods **32**

3.1 Penalized reaction time calculation . . . 32

3.2 Behavioral variability analysis . . . 32

3.3 Anatomical data processing in fMRIPrep . . . 32

3.4 Multilevel community detection for signed networks . . . 32

3.5 Standard GLM analysis . . . 33

References **33**

1 Supplementary Figures

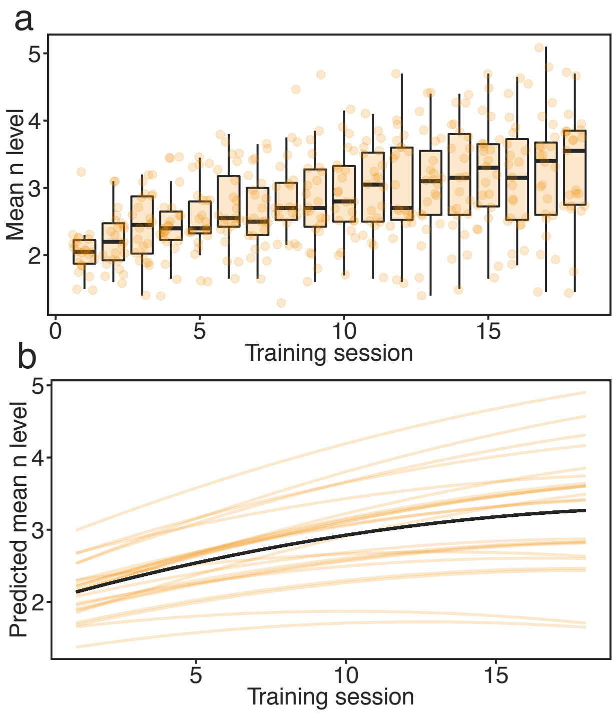

Supplementary Figure 1: **Behavioral performance during dual n-back training.** The performance was measured as a mean n-back level achieved during each trial of 18 training sessions. This measure was estimated only for the experimental group (n = 23). (a) Boxplots represent values of median n-level achieved during 18 sessions of training. Boxes represent the interquartile range (IR) between 25th and 75th percentiles. The upper and lower error bars display the largest and smallest values within 1.5 times IR above 75th percentile and below 25th percentile, respectively.. On average, participants improved their initial performance by 60.3%. Maximum n-back levels achieved by participants varied from 3-back to 7-back. (b) Growth model fitted to mean n-values. We fitted both linear and quadratic models to predict the behavioral score (mean n-back level) monitored across the 18 training sessions. Training session significantly predicted mean n-back level achieved by participants, $\chi^2(2) = 111.21$, $p < 0.0001$. Including a quadratic term in the model based on session significantly improved the model fit, $\chi^2(1) = 24.12$, $p < 0.0001$. Orange lines represent models of behavioral improvement fitted to each participant's performance. The black line represents the prototype model fitted to the experimental group. Source data are provided as a Source Data file.

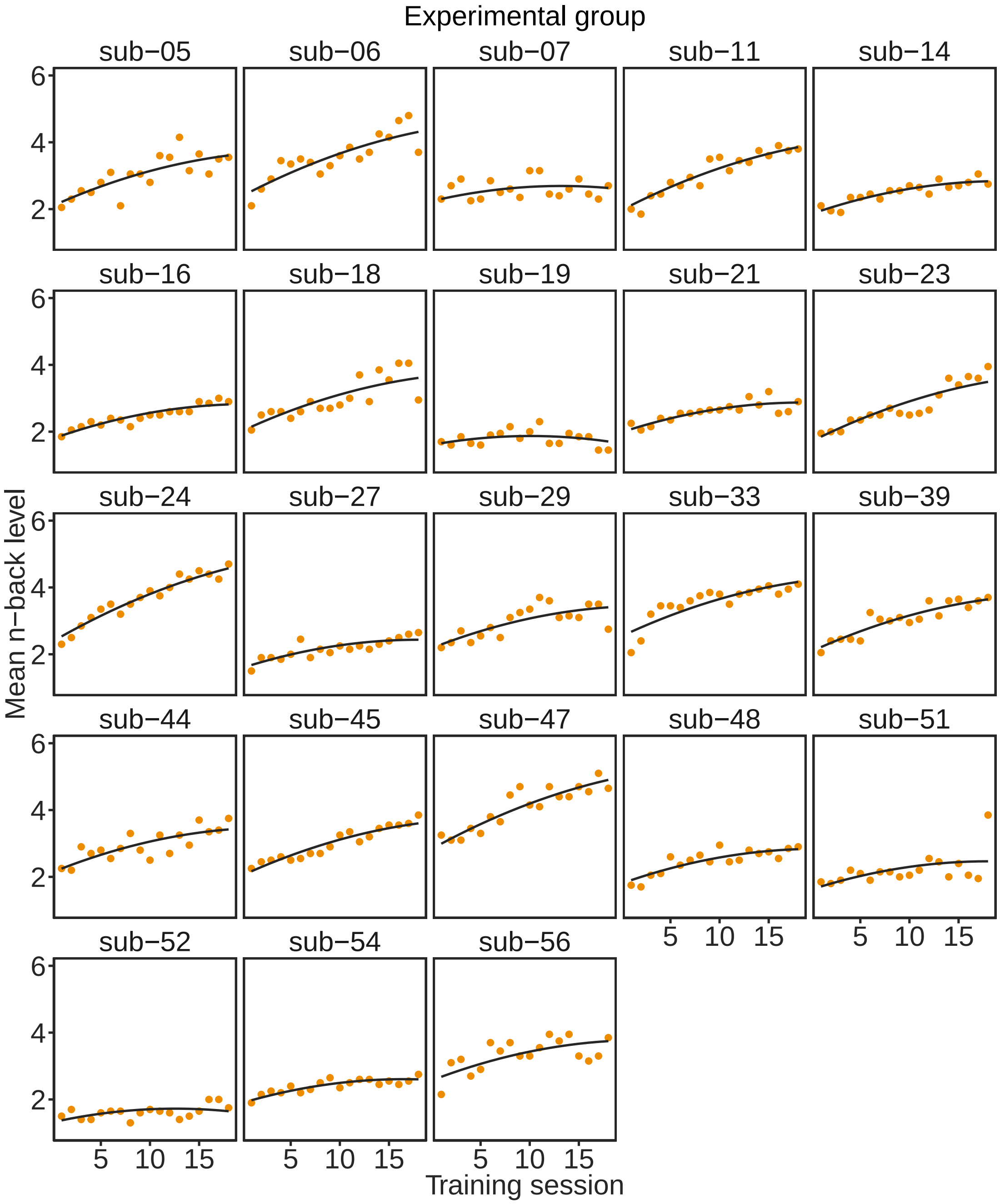

Supplementary Figure 2: **Individual values of mean n-back level achieved in each session of the dual n-back training.** The black line represents a quadratic model fitted to individual data. Source data are provided as a Source Data file.

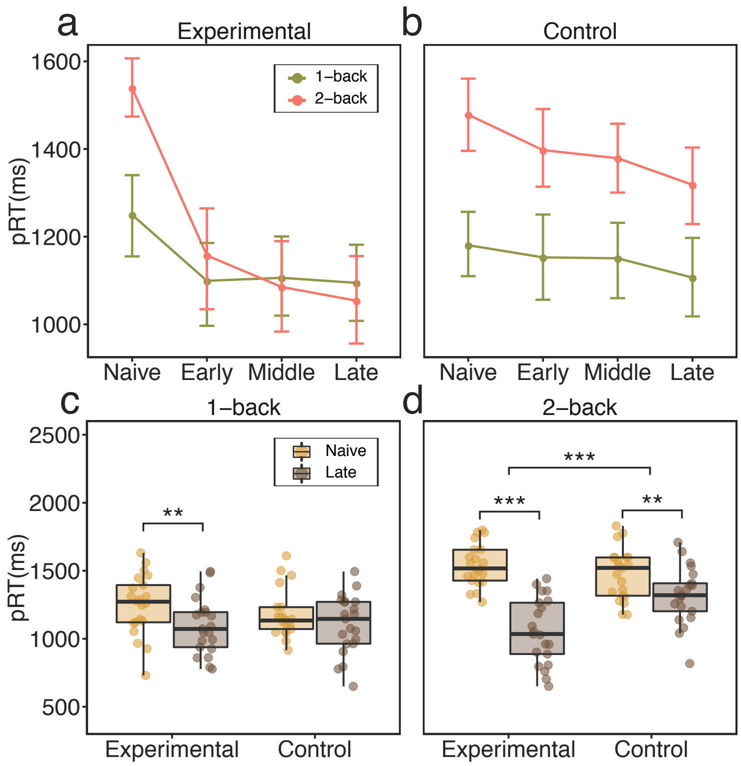

Supplementary Figure 3: **Behavioral performance modulated by training**. (**a, b**) Line plots represent mean behavioral performance measured as pRT, calculated for all training phases (Naive, Early, Middle, Late), dual n-back conditions (1-back and 2-back), and groups (experimental, n = 21 (**a**); control, n = 21 (**b**). Participants exhibited significantly different pRT, depending on the training stage (Naive, Early, Middle, Late), condition (1-back versus 2-back), and group (experimental versus control), as indicated by a χ^2-test ($\chi^2(3) = 21.25$, $p = 0.00009$). Dots represent mean values; error bars represent 95% confidence intervals. (**c**) In the 1-back condition, the experimental group displayed a 14.2% reduction in pRT (paired t-test, two-sided: $t(20) = 3.90$, $p = 0.003$, Bonferroni-corrected); no improvement was found in the control group (paired t-test, two-sided: $t(20) = 1.77$, $p = 0.08$, uncorrected). The change in pRT during the 1-back condition did not differ between the two groups (two-sample t-test, two-sided: $t(39.91) = 1.41$, $p = 0.17$). (d) The greatest improvement was observed in the experimental group when comparing ‘Naive’ to ‘Late’ training phases during the 2-back condition (mean 46 % pRT improvement; paired t-test, two-sided: $t(20) = 10.16$, $p = 0.0003$, Bonferroni-corrected). For comparison, the control group exhibited only a 12.2 % decrease of pRT during the 2-back condition (paired t-test, two-sided: $t(20) = 3.95$, $p = 0.003$, Bonferroni-corrected). The decrease in pRT was significantly larger for the experimental group than for the control group (two-sample t-test, two-sided: $t(38.95) = 5.19$, $p = 0.00001$, Bonferroni-corrected). After training, the experimental group exhibited no difference in performance between the 1-back condition and the 2-back condition after training (paired t-test, two-sided: $t(20) = 1.52$, $p = 0.14$, uncorrected), while in control group, the difference in performance between conditions remained substantial (paired t-test, two-sided: $t(20) = $ -5.71, $p = 0.00002$, Bonferroni-corrected). Boxes represent the interquartile range (IR) between 25th and 75th percentiles. The thick line in the center of each box represents the median. The upper and lower error bars display the largest and smallest values within 1.5 times IR above 75th percentile and below 25th percentile, respectively. *** $p < 0.001$ Bonferroni-corrected; ** $p < 0.01$ Bonferroni-corrected. Source data are provided as a Source Data file.

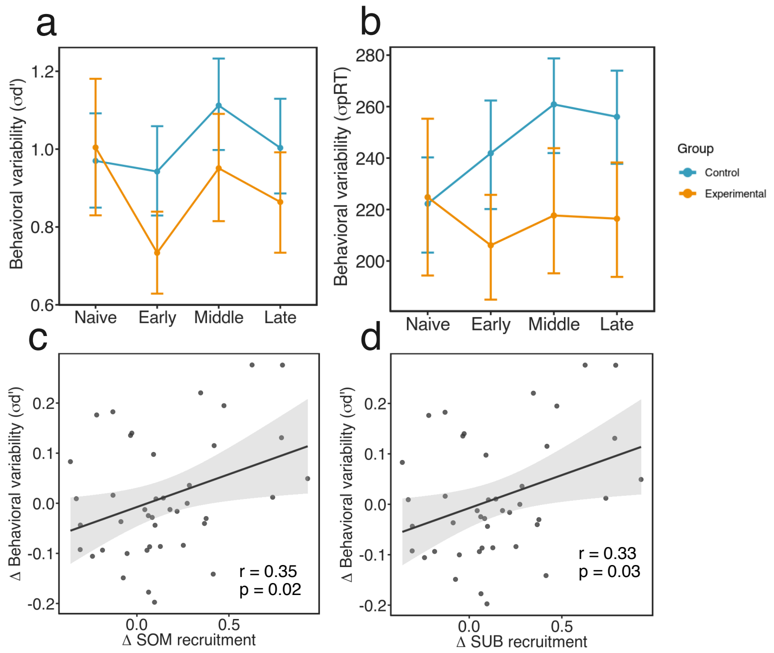

Supplementary Figure 4: **Block-to-block variability in behavioral performance modulated by training**. (**a**) Standard deviation of d' ($\sigma_{d'}$) estimated across task blocks, for which we found a significant main effect of session ($\chi^2(3) = 9.61$, $p = 0.02$). Specifically, the standard deviation of d' decreased from 'Naive' to 'Early' sessions for all participants (paired t-test, two-sided: β = -0.14, $t(39)$ = -2.46, $p = 0.02$; n = 42). Both group and session × group interaction effects were not significant ($p > 0.05$). Dots represent mean values; error bars represent 95% confidence intervals. (**b**) Standard deviation of penalized reaction time (σ_{pRT}), for which we found a significant group effect effect($\chi^2(1) = 7.39$, $p = 0.006$). In general, participants from the experimental group (n = 21) had lower pRT variability (two-sample t-test, two-sided: β = -29.00, $t(40)$ = -2.80, $p = 0.008$) than participants from the control group (n = 21). Both the effect of session and the session × group interaction were not significant ($p > 0.05$). (**c, d**) correlation between the across-session change in behavioral variability measured as standard deviation of d' and the across-session change in (**c**) somatomotor and (d) subcortical systems recruitment ($p < 0.05$, uncorrected). Source data are provided as a Source Data file.

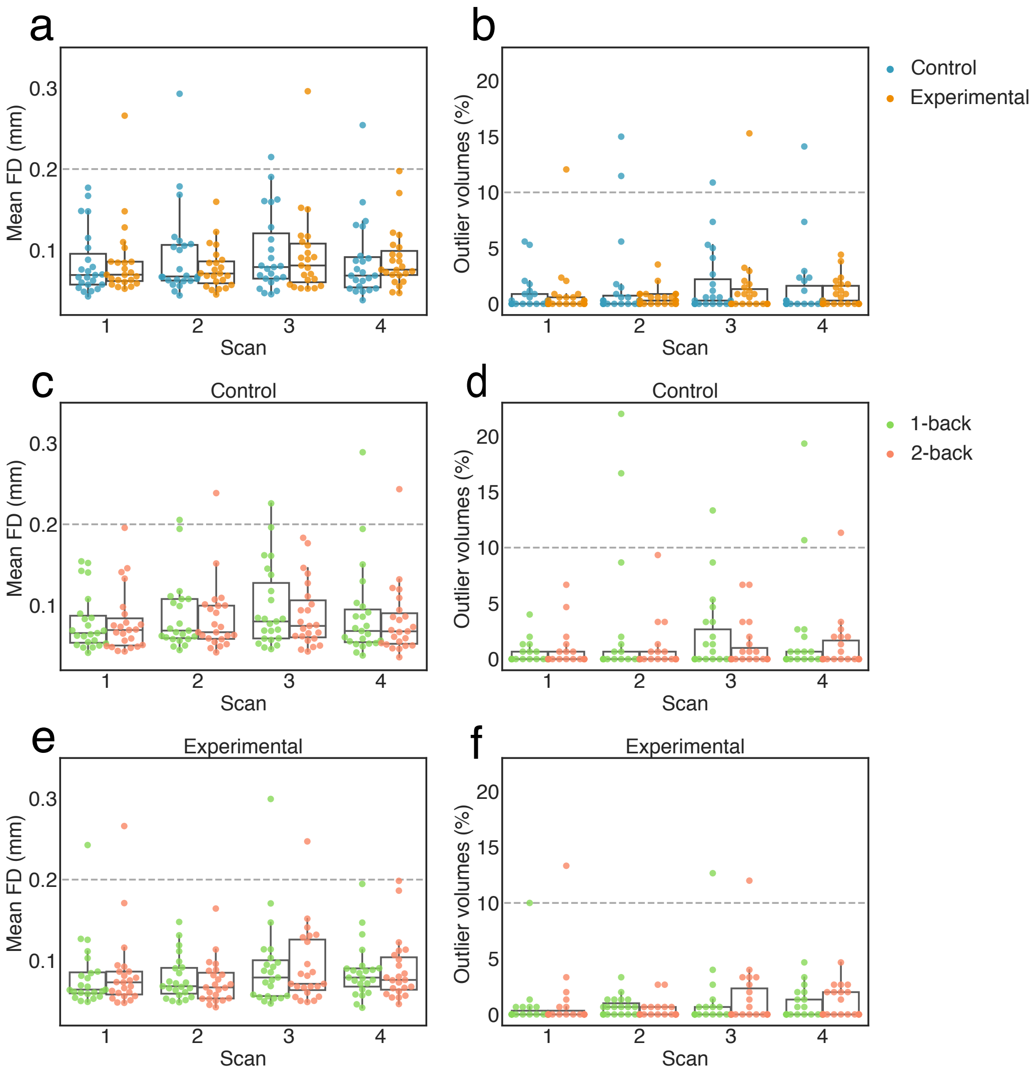

Supplementary Figure 5: **Head motion during the dual n-back task**. In addition to including 24 motion parameters in the denoising procedure, we also excluded high motion subjects from subsequent analyses. We defined a high motion subject as one with mean frame displacement (FD) larger than 0.2 mm (**a, c, e**) and more than 10% of outlier volumes in any scanning session (**b, d, f**). This criterion was applied when considering the total time courses, as well as when considering time courses of the 1-back and 2-back conditions, separately. As a result we excluded four participants (2 from the control group, and 2 from the experimental group) from the participants that completed the training (n = 23 in each group, total n = 46). One subject displayed excessive motion during three scanning sessions, while another displayed excessive motion during two scanning sessions, and two subjects displayed excessive motion in only one scanning session. After excluding high motion subjects, we compared the mean FD and mean percent of outlier scans between sessions, groups, and conditions. We did not find significant differences between any of these variables between sessions (paired-tests, two-sided: all $p < 0.05$ uncorrected), groups (two-sample t-test, two-sided: all < 0.05, uncorrected), and most of the condition comparisons. The only difference that passed an uncorrected threshold of significance (paired t-test, two-sided: $p < 0.05$, uncorrected) was found between the 1-back and 2-back conditions of the control group during the third scanning session. Boxes represent the interquartile range (IR) between 25th and 75th percentiles. The thick line in the center of each box represents the median. The upper and lower error bars display the largest and smallest values within 1.5 times IR above 75th percentile and below 25th percentile, respectively. Source data are provided as a Source Data file.

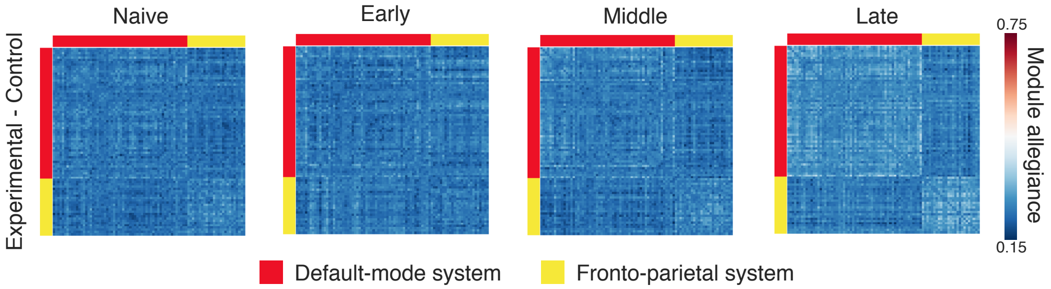

Supplementary Figure 6: **Between-group differences in module allegiance matrices for the default mode and fronto-parietal systems**. Each ij-th element of the matrix represents a difference between groups (experimental minus control) in the probability that node i and node j are assigned to the same module within a single layer of the multilayer network. Systems are defined using the Power et al.[1] parcellation. Source data are provided as a Source Data file.

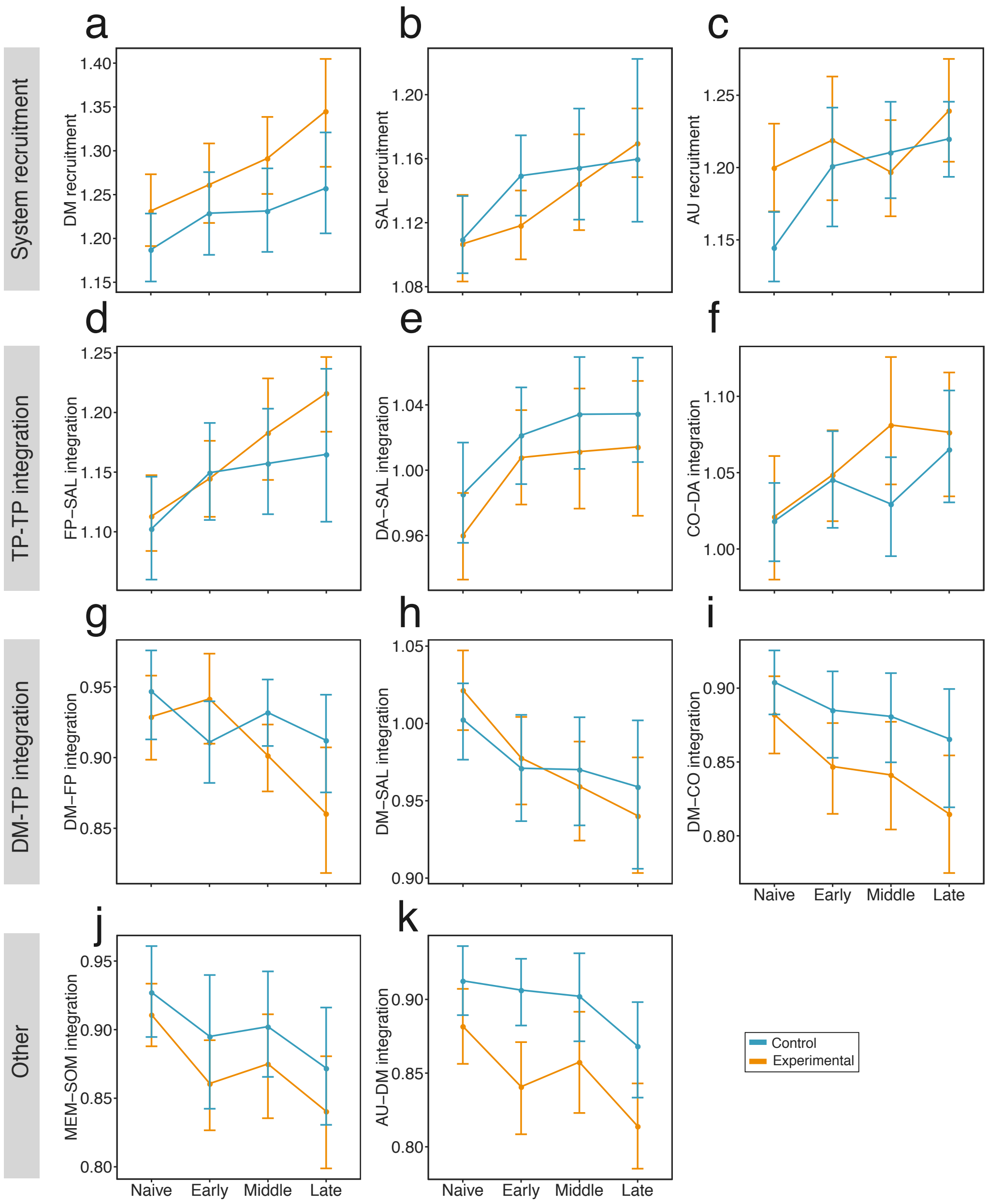

Supplementary Figure 7: **Session-to-session changes in recruitment and integration of large-scale systems**. We observed three main categories of large-scale system reorganization: (**a-c**) an increase in system recruitment, (**d-f**) an increase in integration between task-positive systems (TP), (**g-i**) a decrease in integration between the default mode (DM) system and task-positive systems, and (**j-k**) other. Number of participants in each group: $n = 21$. Dots represent mean values; error bars represent 95% confidence intervals. Remaining abbreviations: salience (SAL), auditory (AU), fronto-parietal (FP), dorsal attention (DA), cingulo-opercular (CO), memory (MEM), and somatomotor (SOM). Source data are provided as a Source Data file.

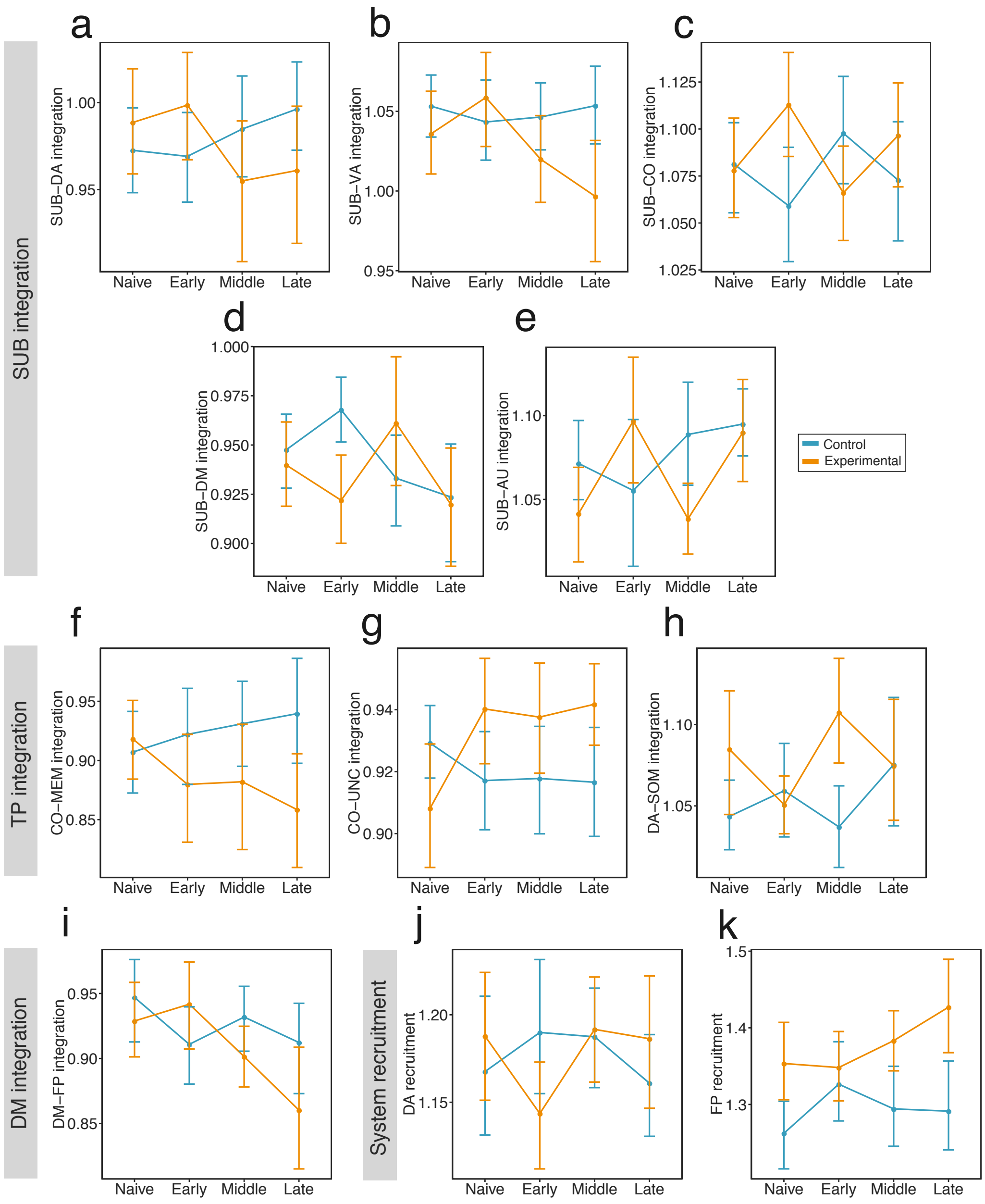

Supplementary Figure 8: **Session × group interaction effects for across-session changes in recruitment** and integration of large-scale systems. We observed group differences in the changes in (**a-e**) integration of subcortical (SUB) system with other systems, (**f-h**) integration of task-positive (TP) systems with other systems, (**i**) integration of the default mode (DM) with the fronto-parietal (FP) system, (**j-k**) changes in dorsal attention (DA) and FP recruitment. Number of participants in each group: n = 21. Dots represent mean values; error bars represent 95% confidence intervals. Remaining abbreviations: salience (SAL), auditory (AU), memory (MEM), and somatomotor (SOM). Source data are provided as a Source Data file.

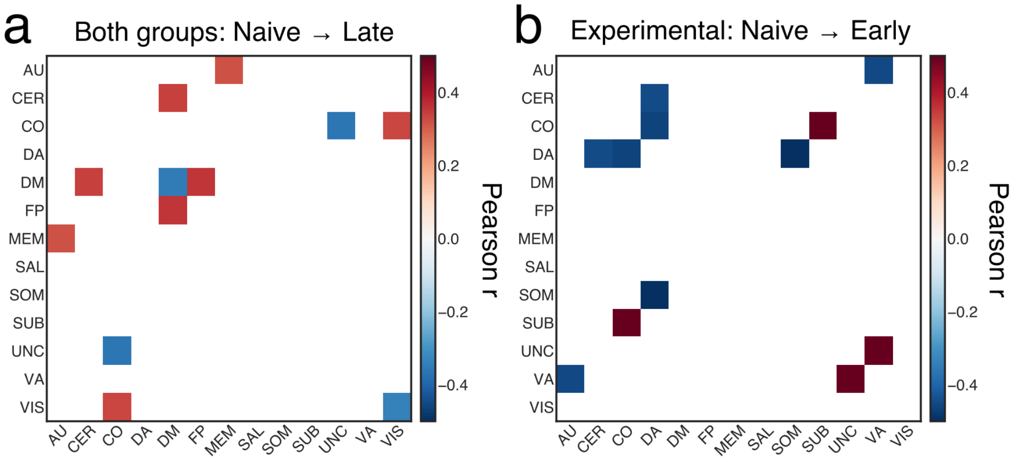

Supplementary Figure 9: **Relationship between the change in network dynamics and the change in behavior**. Colored tiles represent all correlations (p $<$ 0.05, uncorrected; *$p < 0.05$ FDR-corrected). (**a**) Pearson correlation coefficient (r) between the across-session changes in recruitment (or integration) and the across-session changes in penalized reaction time (Δ pRT) observed for both experimental and control group. (**b**) Relationship between the changes in recruitment (or integration) and the changes in pRT during early phase of training of the experimental group. Dots represent mean values; error bars represent 95% confidence intervals. Abbreviations: auditory (AU), cerebellum (CER), cingulo-opercular (CO), default mode (DM), dorsal attention (DA), fronto-parietal (FP), memory (MEM), salience (SAL), somatomotor (SOM), subcortical (SUB), uncertain (UNC), ventral attention (VA), and visual (VIS). Source data are provided as a Source Data file.

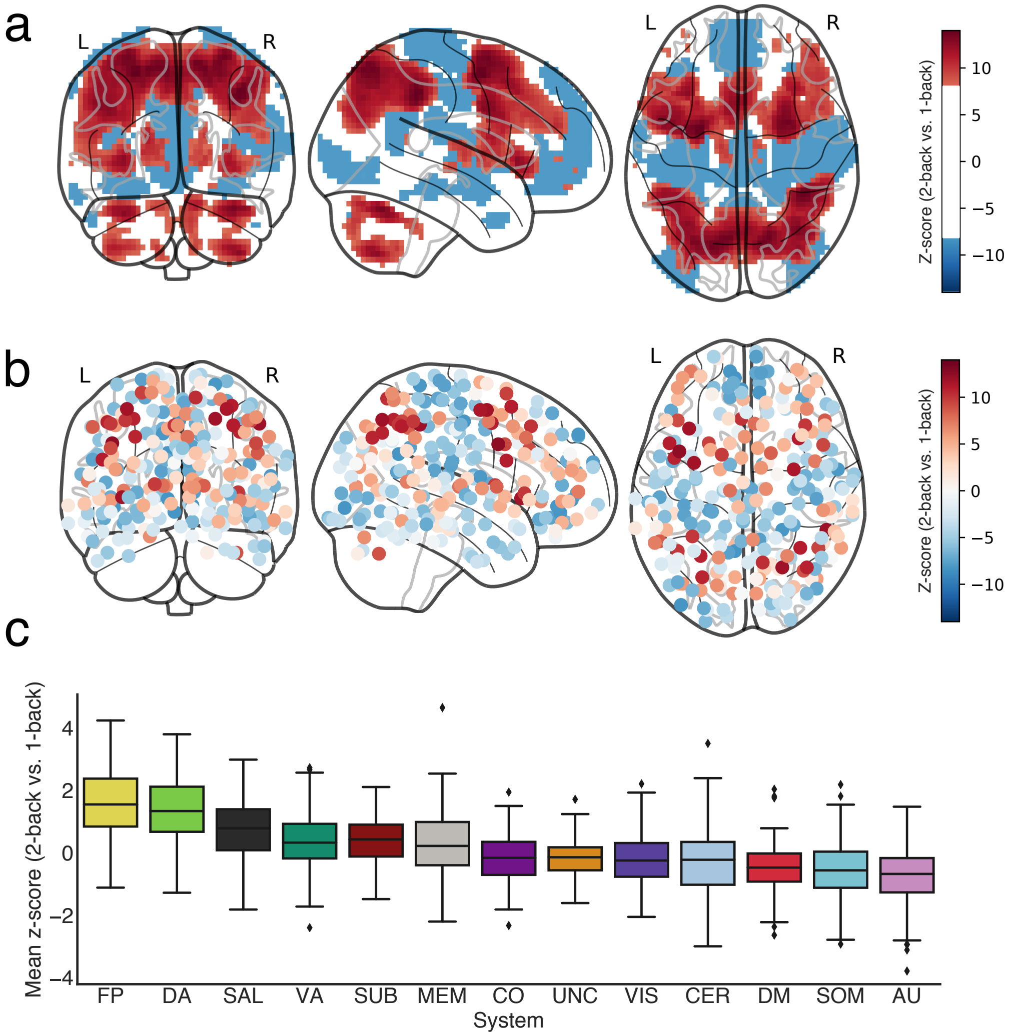

Supplementary Figure 10: **Brain activity for 2-back vs. 1-back contrast (two-sided) estimated with a standard GLM for all subjects and sessions**. (**a**) Glass brain visualization of activity thresholded at a z-score level ± 8. (b) Brain activity plotted on 264 ROIs from the Power et al.[1] parcellation. (**c**) Boxplots representing the median z-score values averaged over ROIs belonging to predefined large-scale systems. Boxes represent the interquartile range (IR) between 25th and 75th percentiles. The upper and lower error bars display the largest and smallest values within 1.5 times IR above 75th percentile and below 25th percentile, respectively. The most active ROIs belonged to the fronto-parietal (FP), dorsal attention (DA), and salience systems (SAL). The most deactivated ROIs belonged to the auditory (AU), somatomotor (SOM), and default mode (DM) systems. Remaining abbreviations: cerebellum (CER), cingulo-opercular (CO), memory (MEM), uncertain (UNC), somatomotor (SOM), subcortical (SUB), ventral attention (VA), and visual (VIS). Source data are provided as a Source Data file. Source data are provided as a Source Data file.

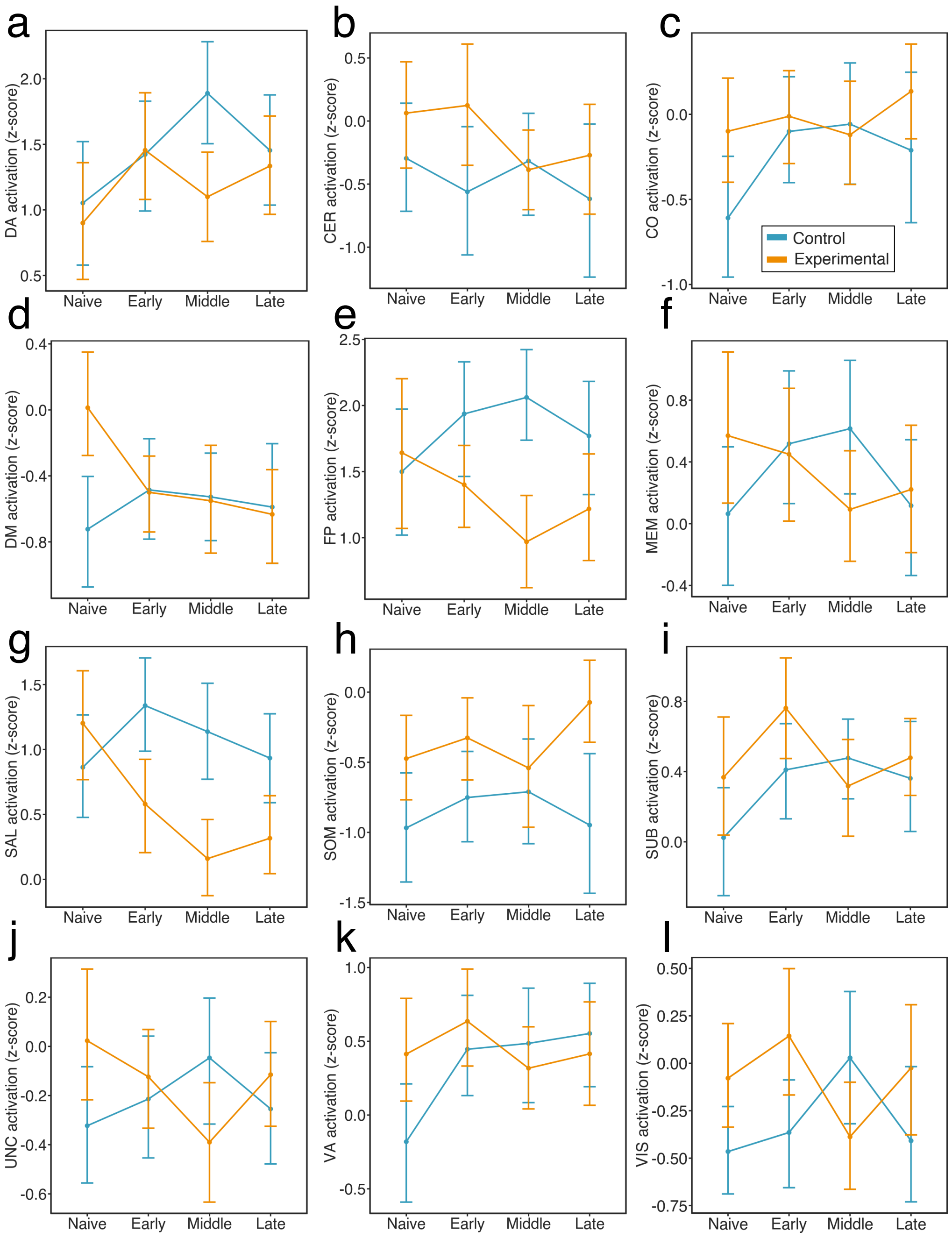

Supplementary Figure 11: **Cross-sessions changes in brain activity for 2-back vs. 1-back contrast (two-sided) estimated with a standard GLM**. Using multilevel modeling, we showed that groups (number of participants in each group: n = 21) differed significantly by session for the salience (SAL) and visual (VIS) systems ($p < 0.05$, FDR-corrected). Specifically, compared to the control group, participants from the experimental group displayed significantly greater decreases in the activation of the salience system from the 'Naive' to 'Early' sessions (two-sample t-test, two-sided: $\beta = -1.10$, $t(120) = -3.44$, $p = 0.0008$), from the 'Naive' to 'Middle' sessions (two-sample t-test, two-sided: $\beta = -1.32$, $t(120) = -4.12$, $p = 0.0001$), and from the 'Naive' to 'Late' sessions (two-sample t-test, two-sided: $\beta = -0.96$, $t(120) = -2.99$, $p = 0.003$). The experimental group also displayed a larger decrease in the activation of the visual system from the 'Naive' to 'Middle' sessions, two-sample t-test, two-sided: $\beta = -0.80$, $t(120) = -2.69$, $p = 0.008$). Dots represent mean values; error bars represent 95% confidence intervals. Source data are provided as a Source Data file.

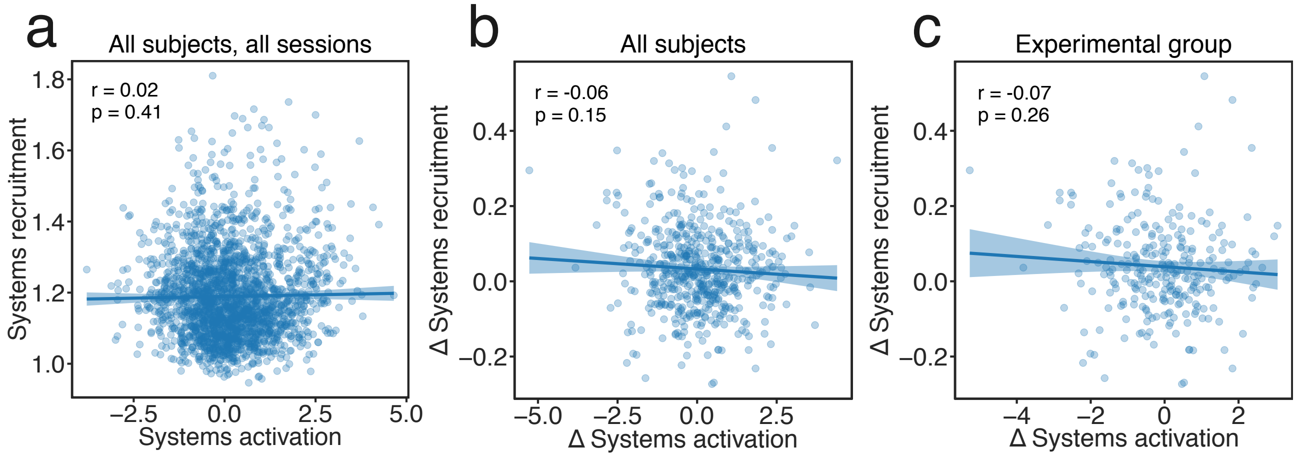

Supplementary Figure 12: **Relationship between systems recruitment and systems activation estimates**. (**a**) There was no significant relationship, measured as Pearson correlation (r), between systems activation (z-score; 2-back minus 1 back) and systems recruitment values when considering all systems, all sessions, and all subjects. We further tested whether changes (δ) in systems activity from 'Naive' to 'Late' sessions were correlated with changes in systems recruitment. We did not find any significant correlation between these two variables, either when considering (**b**) all subjects, or (**c**) when considering only the experimental group. Source data are provided as a Source Data file.

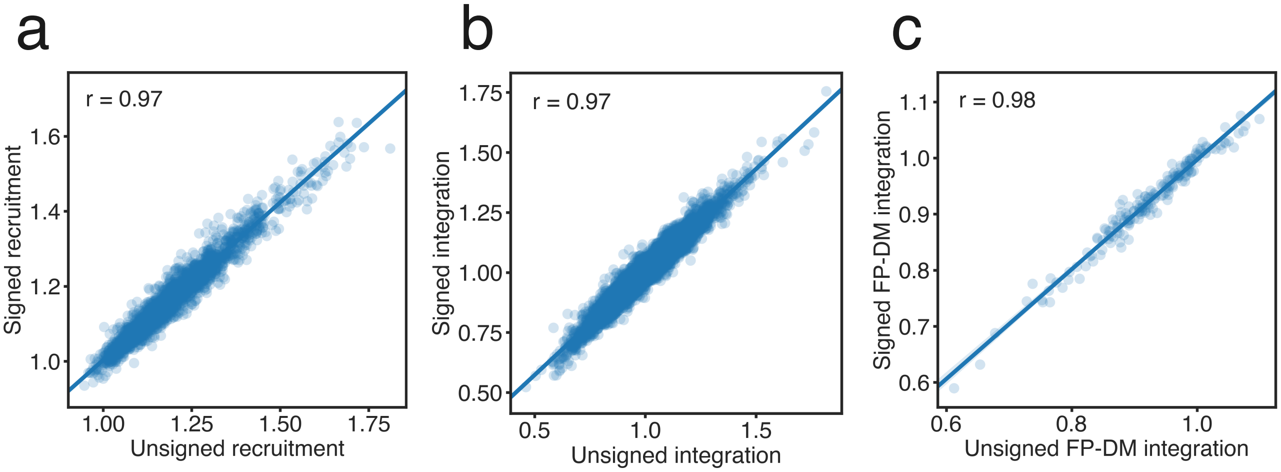

Supplementary Figure 13: **Relationship between recruitment and integration values calculated based on unsigned and signed functional connectivity matrices**. Unsigned and signed recruitment (**a**) and integration (**b**) coefficients estimated for all large-scale systems were highly correlated, as measured with Pearson correlation (r). (**c**) Values of integration between fronto-parietal (FP) and default mode (DM) systems were also highly correlated. Source data are provided as a Source Data file.

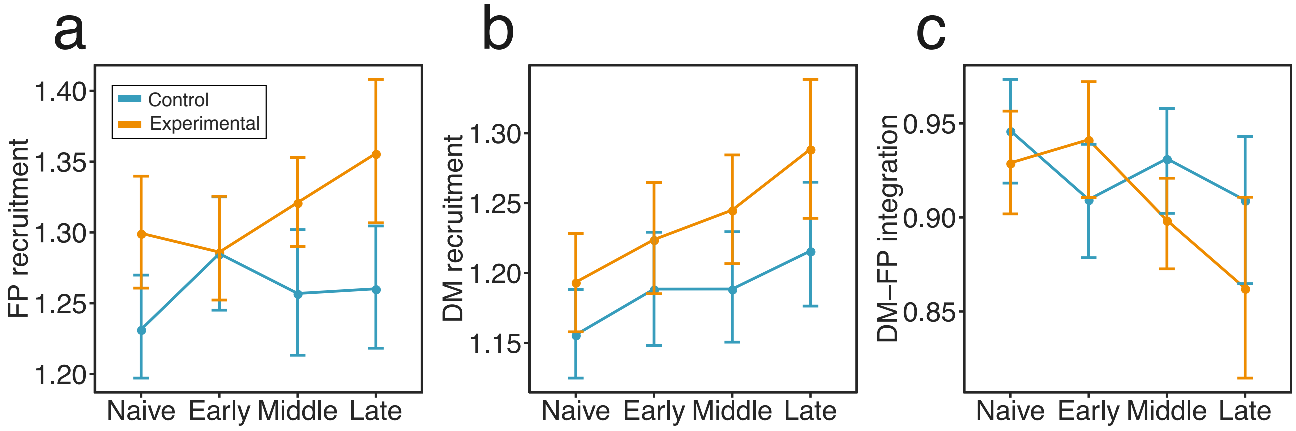

Supplementary Figure 14: **Changes in module allegiance of the fronto-parietal (FP) and default-mode (DM) systems calculated based on signed functional connectivity matrices**. We observed a significant session × group interaction effect (number of participants in each group: n = 21) when considering changes in the recruitment of the fronto-parietal system during training ($\chi^2(3) = 9.31$, $p = 0.025$) (a). The largest increase in fronto-parietal recruitment was observed in the experimental group when comparing 'Early' to 'Late' training phases (β = -0.07, $t(120)$ = -3.057, $p = 0.016$, Bonferroni-corrected). No significant changes from 'Naive' to 'Late' training phases were observed in the control group (β = -0.05, $t(120)$ = -2.35, $\beta = 0.12$, Bonferroni-corrected). (**b**) Turning to an examination of the default mode, we found a significant main effect of session ($\chi^2(3) = 23.89$, $p < 0.0001$) on system recruitment However, the interaction effect between session and group was not significant ($\chi^2(3) = 2.00$, $p = 0.57$). Planned contrasts revealed that the default mode recruitment increased steadily in both groups and we observed the largest increase between 'Naive' and 'Late' sessions ($\beta = 0.08$, $t(123) = 5.02$, $p < 0.0001$). (**c**) We found a significant session × group interaction effect on the integration between the fronto-parietal and default mode systems ($\chi^2(3) = 13.30$, $p = 0.004$). The integration between these two systems decreased from 'Early' to 'Late' sessions only in the experimental group ($\beta = 0.08$, $t(120) = 4.86$, $p = 0.0035$, Bonferroni-corrected). However, groups differed from 'Naive' to 'Early' ($\beta = 0.05$, $t(120) = 2.13$, $p = 0.03$) and from 'Early' to 'Middle' sessions (β= -0.06, $t(120)$ = -2.81, $p = 0.02$. Dots represent mean values; error bars represent 95% confidence intervals. Source data are provided as a Source Data file.

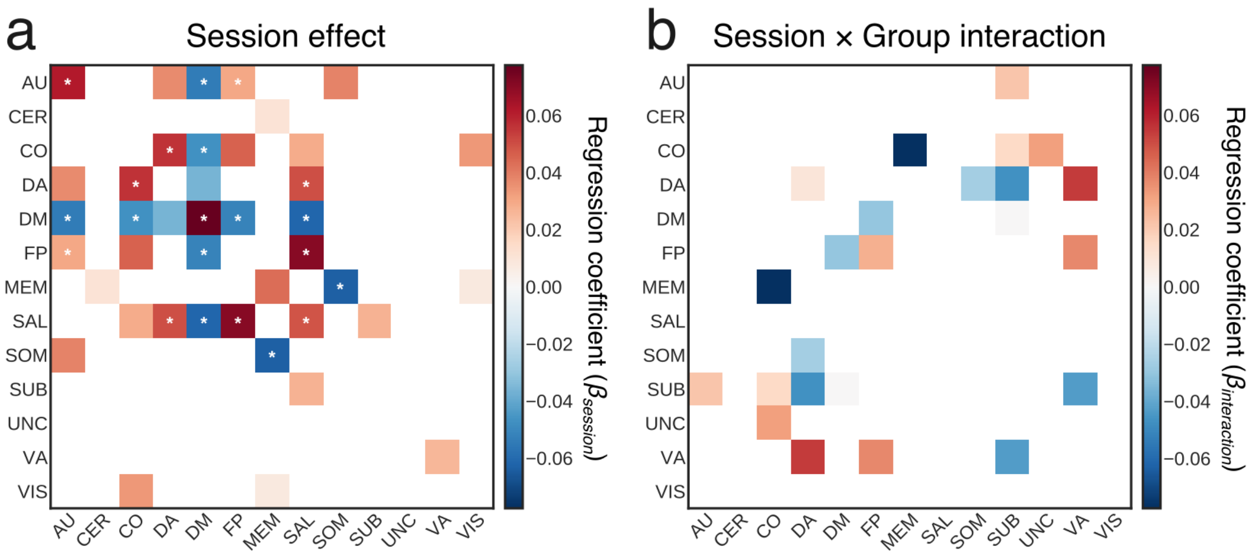

Supplementary Figure 15: **Changes of the recruitment and integration of large-scale systems calculated based on signed functional connectivity matrices**. Colored tiles represent all significant effects ($p < 0.05$, uncorrected; *$p < 0.05$ FDR-corrected). (**a**) Here we display the significant main effects of session. Tile color codes a linear regression coefficient (β), for all main session effects (from 'Naive' to 'Late'). (**b**) Here we display the significant session × group interaction effects. Tile color codes a linear regression coefficient between groups and sessions (from 'Naive' to 'Late'). Abbreviations: auditory (AU), cerebellum (CER), cingulo-opercular (CO), default mode (DM), dorsal attention (DA), fronto-parietal (FP), memory (MEM), salience (SAL), somatomotor (SOM), subcortical (SUB), uncertain (UNC), ventral attention (VA), and visual (VIS). Source data are provided as a Source Data file.

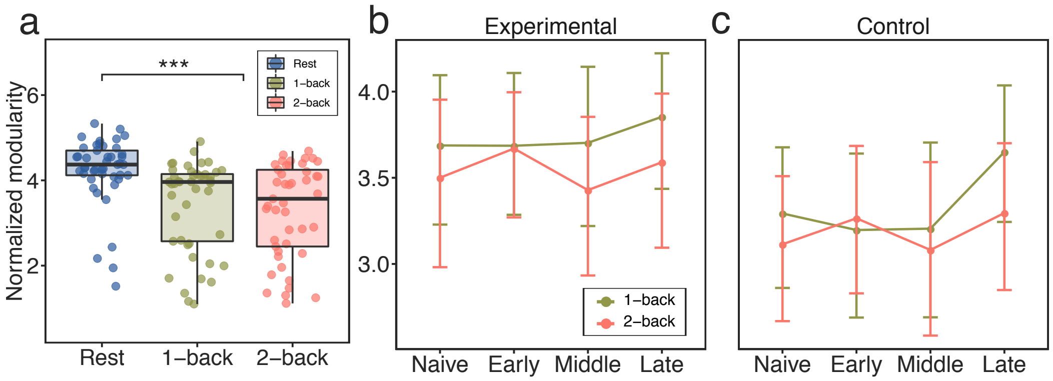

Supplementary Figure 16: **Whole-brain modularity obtained for the Schaefer parcellation.** (a). Modularity differences between the resting state and the dual n-back task, as well as between the 1-back task condition and the 2-back task condition estimated during the first scanning session for all subjects (paired t-tests, n = 46). Boxes represent the interquartile range (IR) between 25th and 75th percentiles. The thick line in the center of each box represents the median. The upper and lower error bars display the largest and smallest values within 1.5 times IR above 75th percentile and below 25th percentile, respectively. (**b, c**) Line plots representing mean values of modularity for each scanning session (Naive, Late, Middle, Late) and condition, separately for (b) the experimental group (n = 21) and for (c) the control group (n = 21). Dots represent mean values; error bars represent 95% confidence intervals. Source data are provided as a Source Data file.

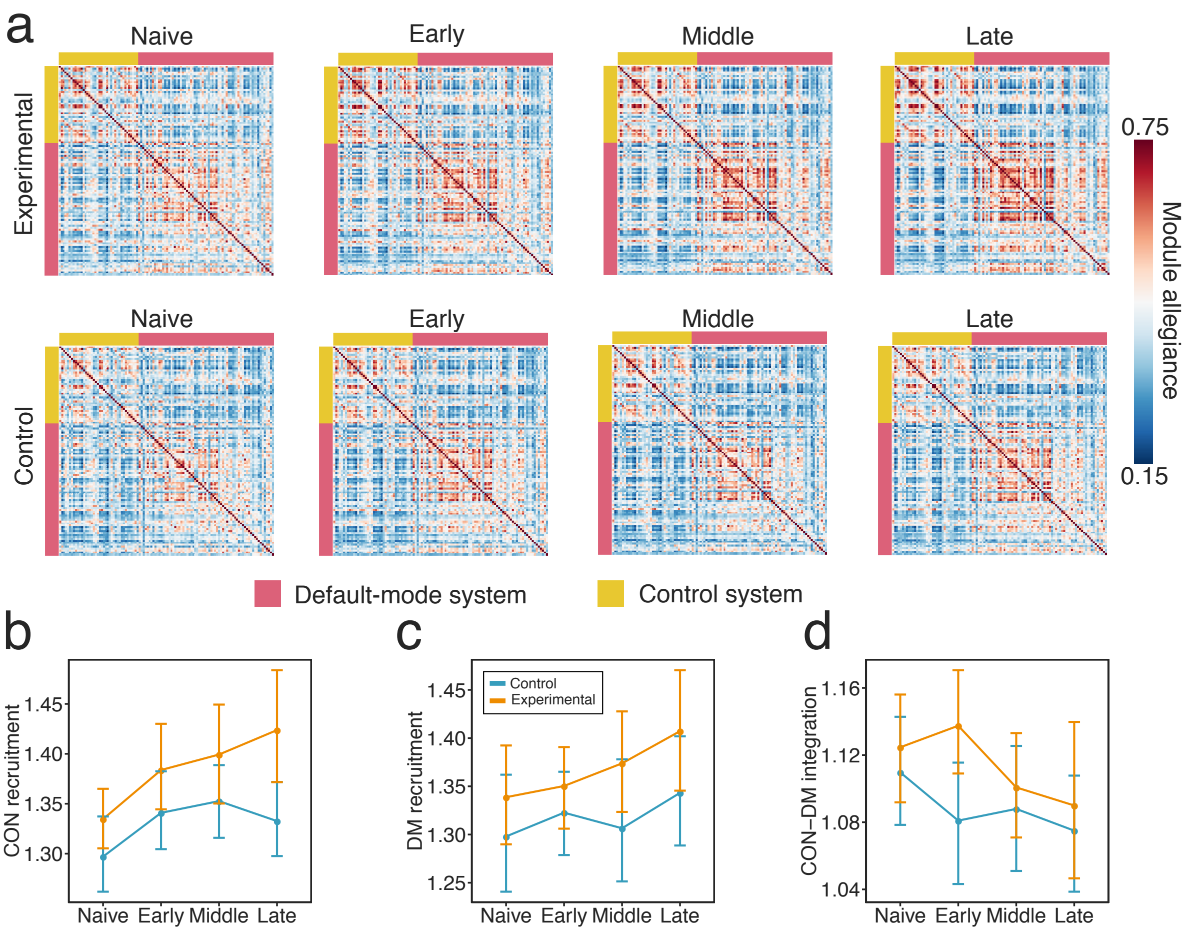

Supplementary Figure 17: **Training-related changes in module allegiance for the subgraph of the network composed of the default mode and fronto-parietal control (CON) systems calculated using the Schaefer parcellation.** (**a**) Module allegiance matrices of the default mode system and the fronto-parietal control system (CON). Each ij-th element of the module allegiance matrix represents the probability that node i and node j are assigned to the same community within a single layer of the multilayer network representing task conditions pooled across all scanning sessions. (**b**) Mean CON recruitment across sessions. (**c**) Mean default mode system recruitment across sessions. (**d**) Mean integration between the default mode and CON systems across sessions. Only CON recruitment exhibited a significant main effect of session ($p < 0.002$). Number of participants in each group: n = 21. Dots represent mean values; error bars represent 95% confidence intervals. Source data are provided as a Source Data file.

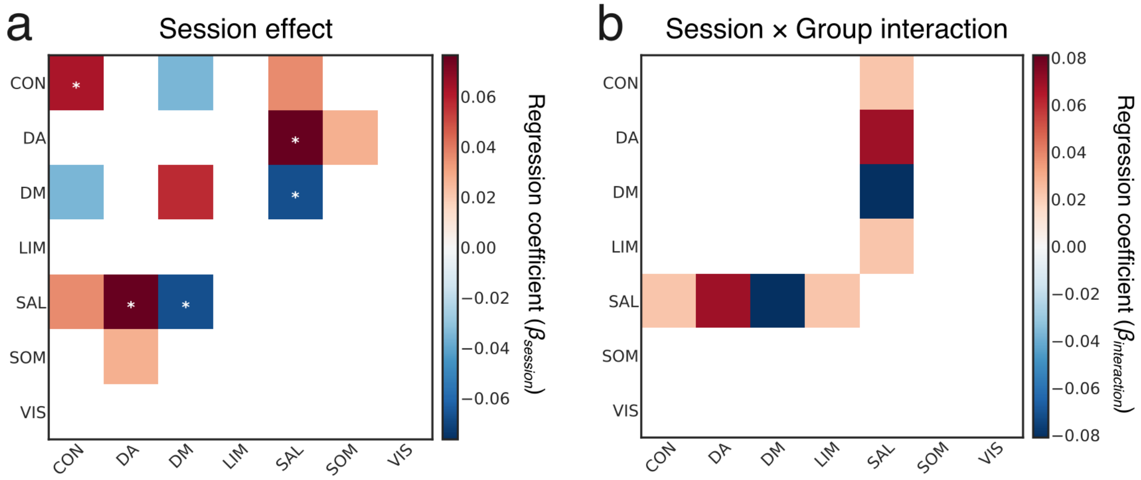

Supplementary Figure 18: **Changes of the recruitment and integration of large-scale systems calculated for Schaefer parcellation [2]**. Colored tiles represent all significant effects ($p < 0.05$, uncorrected; $*p < 0.05$ FDR-corrected). (**a**) Here we display the significant main effects of session. Tile color codes a linear regression coefficient (β), for all main session effects (from 'Naive' to 'Late'). (**b**) Here we display the significant session $\times$ group interaction effects. Tile color codes a linear regression coefficient between groups and sessions (from 'Naive' to 'Late'). Abbreviations: control (CON), dorsal attention (DA), default mode (DM), limbic (LIM), salience (SAL), somatomotor (SOM), visual (VIS). Source data are provided as a Source Data file.

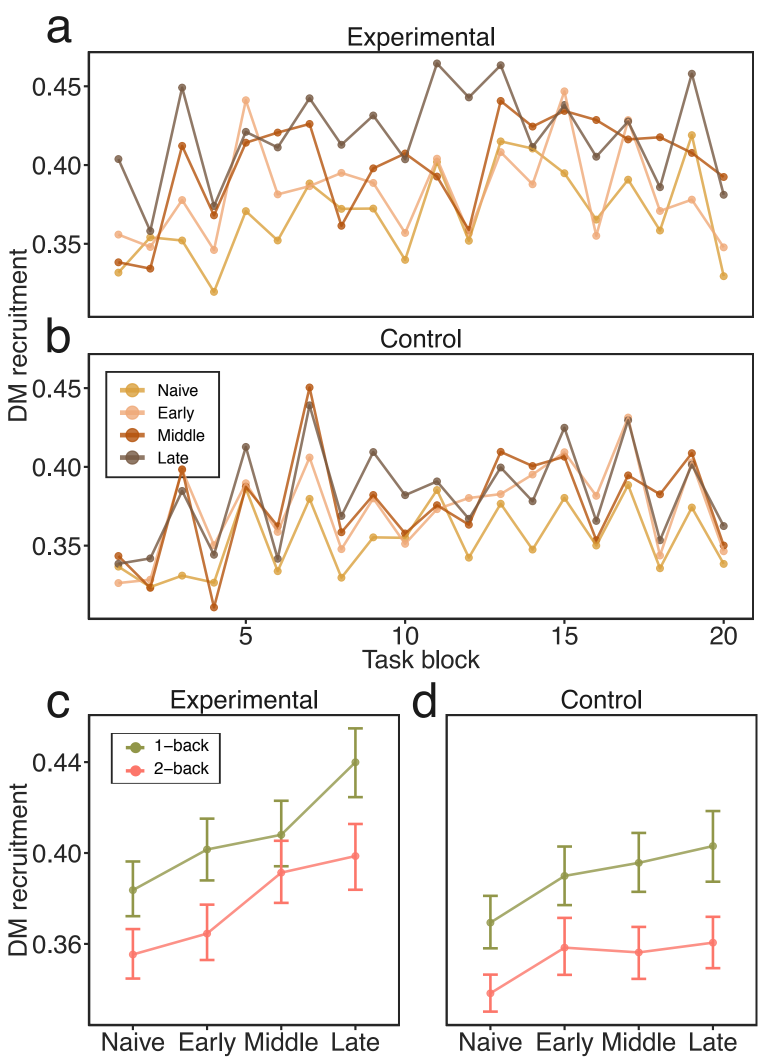

Supplementary Figure 19: **Fluctuations in the recruitment of the default mode system across task blocks**. We examined changes in the default mode recruitment by calculating allegiance matrices for each task block. We found a significant effect of condition ($\chi^2(3) = 83.97$, $p < 0.00001$), such that the recruitment of the default mode fluctuated between task conditions and was significantly higher in the 1-back condition (M = 0.40) than in the 2-back condition (M = 0.36; paired t-test, two-sided: $t(167) = -10.43$, $p < 0.00001$). However, the session × condition interaction was not significant ($\chi^2(3) = 2.82$, $p = 0.40$). Collectively, these results suggest that the default mode recruitment is not only modulated by working memory training, but also by the changing demands of the cognitive task. Across-block fluctuations in default mode recruitment in (**a**) the experimental group and (**b**) the control group. Differences between task conditions for (**c**) the experimental group and (**d**) the control group, across training stages. Dots represent mean values; error bars represent 95% confidence intervals. Source data are provided as a Source Data file.

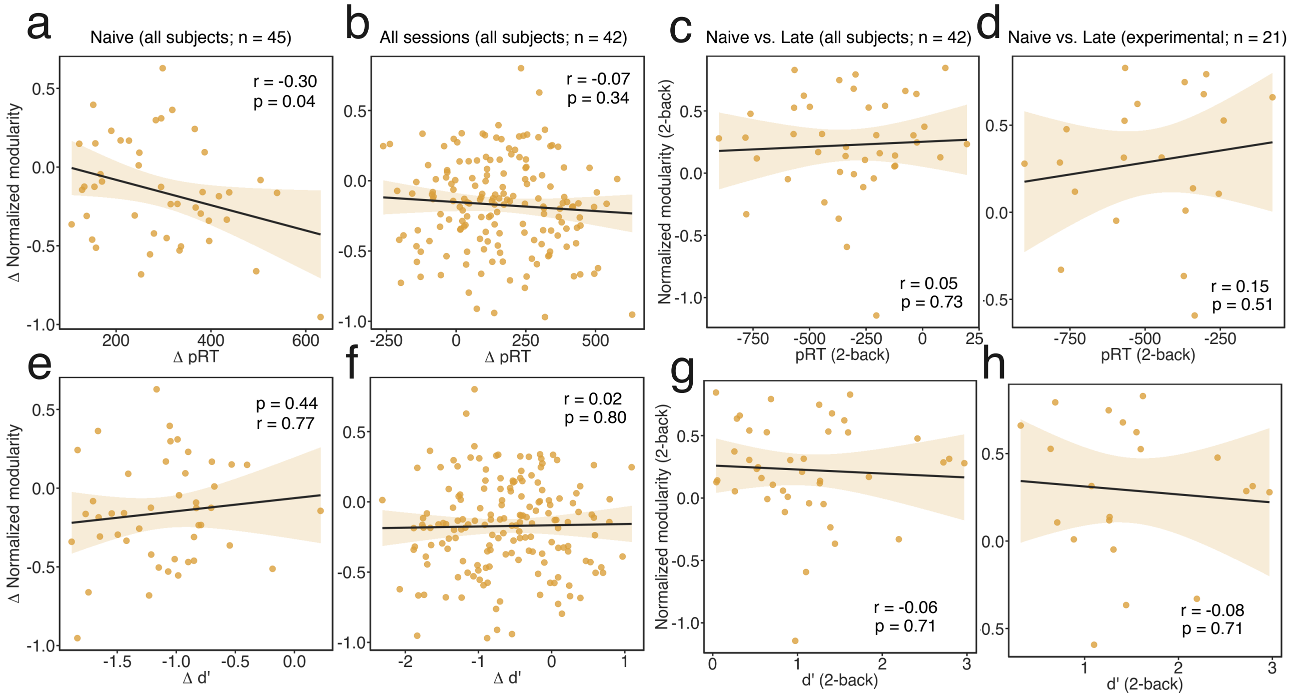

Supplementary Figure 20: **Relationship between modularity and behavioral performance**. (a) We observed a weak negative correlation between the change (Δ) of modularity (2-back - 1-back) and the change in penalized reaction time (Δ pRT) during 'Naive' session. (b) Change of modularity was not related to the changes in pRT when considered all scanning sessions. (e, f) We did not observe any relationship between the change in d' and the change in modularity for 'Naive' and for all scanning sessions. (c, d, g, h) The change of modularity during 2-back was not correlated to the changes in pRT or d' from 'Naive' to 'Late' session.

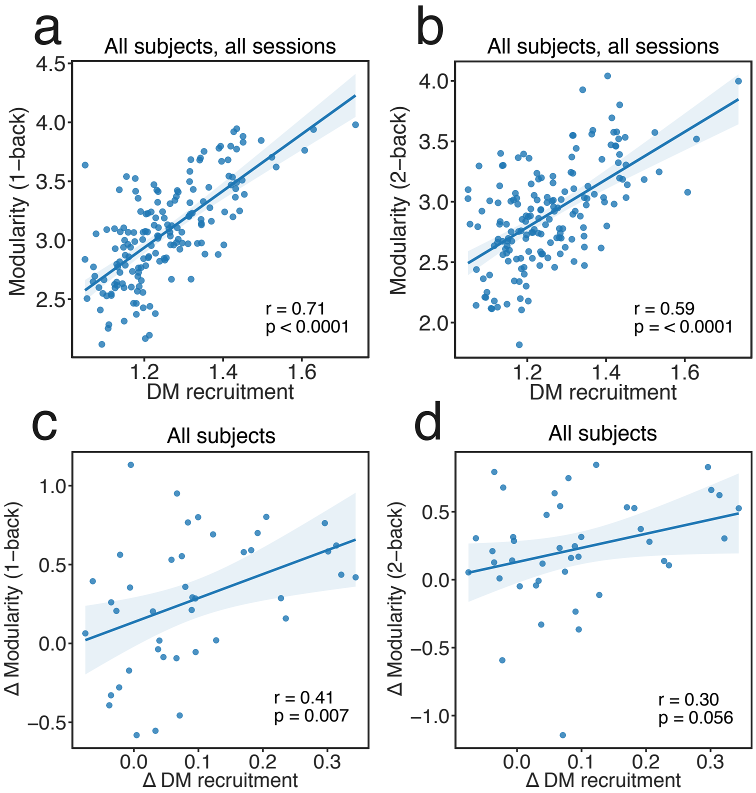

Supplementary Figure 21: **Relationship between default mode recruitment and static modularity**. Correlation between DM recruitment and modularity during (**a**) 1-back and (b) 2-back conditions calculated for all subjects and all sessions. Correlation between the change (Δ) of DM recruitment and change of modularity during (c) 1-back condition and (**d**) 2-back condition. Source data are provided as a Source Data file.

2 Supplementary Tables

**Repeated measures MLM: main effect of session**			
Systems	Statistics		
	χ^2	$p_{uncorr.}$	p_{FDR}
Recruitment			
SAL	24.3038	0.0000	0.0005
DM	24.1711	0.0000	0.0005
AU	14.7401	0.0021	0.0208
MEM	9.8965	0.0195	0.1181
SUB	8.5157	0.0365	0.1747
Integration with default mode (DM) system			
DM-SAL	31.3720	0.0000	0.0001
DM-AU	20.1747	0.0002	0.0024
DM-FP	19.7550	0.0002	0.0025
DM-CO	15.8257	0.0012	0.0140
Integration with task-positive systems			
FP-SAL	28.8229	0.0000	0.0001
DA-SAL	21.8571	0.0001	0.0013
CO-DA	14.0292	0.0029	0.0261
MEM-SOM	13.1715	0.0043	0.0354
SAL-MEM	11.0954	0.0112	0.0785
SAL-SUB	10.0385	0.0182	0.1181
FP-CO	9.7186	0.0211	0.1201
CO-VIS	9.499441	0.023337	0.124923
SAL-CO	8.9760	0.0296	0.1497
FP-AU	8.2521	0.0411	0.1869
VA-UNC	7.9361	0.0474	0.2032
Other			
AU-SOM	11.6143	0.0088	0.0669

Supplementary Table 1: **Results of the multilevel modeling (MLM) analysis reflecting main session effects for systems recruitment or integration (4 sessions)**. In all cases, random intercepts were estimated. The significance of models was estimated with chi-square tests, where models with increasing complexity were compared and the resulting value of Likelihood Ratio Test (χ^2) and corresponding p-value (uncorrected and FDR-corrected) were reported[3]. Abbreviations: auditory (AU), cerebellum (CER), cingulo-opercular (CO), default mode (DM), dorsal attention (DA), fronto-parietal (FP), memory (MEM), salience (SAL), somatomotor (SOM), subcortical (SUB), uncertain (UNC), ventral attention (VA), and visual (VIS).

**Planned contrasts: main effect of session**						
Systems	Naive vs. Early		Naive vs. Middle		Naive vs. Late	
	β	p	β	p	β	p
Recruitment						
SAL	0.0259	0.0279	0.041229	0.0006	0.057214	0.0000
DM	0.0358	0.0528	0.052197	0.0051	0.091959	0.0000
AU	0.0377	0.0127	0.0314	0.0369	0.0572	0.0002
MEM	0.0292	0.1145	0.016409	0.3740	0.05636	0.0027
SUB	0.0409	0.0145	0.015256	0.3569	0.039226	0.0190
Integration with default mode (DM) system						
DM-SAL	-0.0378	0.0008	-0.047378	0.0000	-0.0623	0.0000
DM-AU	-0.0237	0.0579	-0.0174	0.1633	-0.0560	0.0000
DM-FP	-0.0116	0.3310	-0.021237	0.0758	-0.051636	0.0000
DM-CO	-0.0273	0.0399	-0.0323	0.0154	-0.0529	0.0001
Integration with task-positive systems						
FP-SAL	0.0395	0.0120	0.062749	0.0001	0.082824	0.0000
DA-SAL	0.0420	0.0009	0.05039	0.0001	0.051894	0.0001
CO-DA	0.0271	0.0512	0.0356	0.0109	0.0511	0.0003
MEM-SOM	-0.0408	0.0201	-0.030599	0.0798	-0.062644	0.0004
SAL-MEM	0.0109	0.4989	0.015714	0.3298	0.050995	0.0019
SAL-SUB	0.0364	0.0037	0.029535	0.0179	0.027495	0.0272
FP-CO	0.0276	0.0736	0.0349	0.0240	0.0459	0.0032
CO-VIS	0.024047	0.0535	0.022812	0.0668	0.037705	0.0027
SAL-CO	0.0257	0.0152	0.0192	0.0683	0.0285	0.0072
FP-AU	-0.0167	0.1912	-0.0025	0.8442	0.0196	0.1242
VA-UNC	-0.0103	0.1671	-0.020728	0.0059	-0.008145	0.2726
Other						
AU-SOM	0.0407	0.0056	0.0337	0.0213	0.0446	0.0025

Supplementary Table 2: **Planned contrasts for all significant main session effects, reflecting changes of systems recruitment or integration (4 sessions)**. Contrasts (paired t-tests, two-sided): 'Naive' vs. 'Early', 'Naive' vs. 'Middle', 'Naive' vs. 'Late'. Abbreviations: auditory (AU), cerebellum (CER), cingulo-opercular (CO), default mode (DM), dorsal attention (DA), fronto-parietal (FP), memory (MEM), salience (SAL), somatomotor (SOM), subcortical (SUB), uncertain (UNC), ventral attention (VA), and visual (VIS).

**Repeated measures MLM: session × group interaction**			
Systems	Statistics		
	χ^2	$p_{uncorr.}$	p_{FDR}
Recruitment			
FP	6.831	0.029	0.327
DA	2.697	0.041	0.339
Integration with default mode (DM) system			
DM-FP	19.755	0.003	0.235
Integration with subcortical (SUB) system			
SUB-DM	5.999	0.015	0.278
SUB-AU	5.843	0.02	0.309
SUB-VA	4.868	0.037	0.334
SUB-DA	1.193	0.026	0.327
SUB-CO	0.257	0.011	0.244
Integration with other task-positive systems			
DA-SOM	2.514	0.011	0.244
CO-MEM	0.865	0.034	0.334
CO-UNC	2.437	0.005	0.236

Supplementary Table 3: **Results of the multilevel modeling (MLM) analysis reflecting session × group interaction effects for systems recruitment or integration (4 sessions, 2 groups)**. In all cases, random intercepts were estimated. The significance of models was estimated with chi-square tests, where models with increasing complexity were compared and the resulting value of Likelihood Ratio Test (χ^2) and corresponding p-value (uncorrected and FDR-corrected) were reported[3]. Abbreviations: auditory (AU), cerebellum (CER), cingulo-opercular (CO), default mode (DM), dorsal attention (DA), fronto-parietal (FP), memory (MEM), salience (SAL), somatomotor (SOM), subcortical (SUB), uncertain (UNC), ventral attention (VA), and visual (VIS).

**Planned contrasts: session × group interaction**						
Systems	Naive vs. Early		Naive vs. Middle		Naive vs. Late	
	β	p	β	p	β	p
Recruitment						
FP	-0.0690	0.0740	-0.0020	0.9570	0.0450	0.2490
DA	-0.0670	0.0200	-0.0160	0.5700	0.0050	0.8560
Integration with default mode (DM) system						
DM-FP	0.0490	0.0330	-0.0120	0.5890	-0.0340	0.1380
Integration with subcortical (SUB) system						
SUB-DM	-0.0380	0.1010	0.0360	0.1230	0.0040	0.8630
SUB-AU	0.0720	0.0230	-0.0200	0.5150	0.0250	0.4250
SUB-VA	0.0330	0.1970	-0.0090	0.7180	-0.0400	0.1180
SUB-DA	0.0140	0.6090	-0.0460	0.0850	-0.0510	0.0540
SUB-CO	0.0570	0.0350	-0.0280	0.2940	0.0270	0.3140
Integration with other task-positive systems						
DA-SOM	-0.0500	0.0690	0.0290	0.2850	-0.0420	0.1250
CO-MEM	-0.0530	0.0980	-0.0600	0.0630	-0.0920	0.0050
CO-UNC	0.0440	0.0040	0.0410	0.0080	0.0460	0.0030

Supplementary Table 4: **Planned contrasts for all significant session × group interaction effects, reflecting group differences in changes of systems recruitment or integration (4 sessions, 2 groups)**. Contrasts (two-sample t-tests, two-sided) for differences: 'Naive' vs. 'Early', 'Naive' vs. 'Middle', 'Naive' vs. 'Late'. Abbreviations: auditory (AU), cerebellum (CER), cingulo-opercular (CO), default mode (DM), dorsal attention (DA), fronto-parietal (FP), memory (MEM), salience (SAL), somatomotor (SOM), subcortical (SUB), uncertain (UNC), ventral attention (VA), and visual (VIS).

**Post-hoc tests: session × group interaction**				
Systems	Early vs. Middle		Middle vs. Late	
	β	$p_{bonferroni}$	β	$p_{bonferroni}$
Recruitment				
FP	0.0673	0.2499	0.0467	0.6842
DA	0.0506	0.2245	0.0212	1.0000
Integration with default mode (DM) system				
DM-FP	-0.0613	0.0237	-0.0216	1.0000
Integration with subcortical (SUB) system				
SUB-DM	0.0739	0.0052	-0.0318	0.5121
SUB-AU	-0.0919	0.0114	0.0453	0.4462
SUB-VA	-0.0418	0.2997	-0.0305	0.6836
SUB-DA	-0.0593	0.0786	-0.0055	1.0000
SUB-CO	-0.0854	0.0055	0.0554	0.1229
Integration with other task-positive systems				
DA-SOM	0.0790	0.0130	-0.0711	0.0300
CO-MEM	-0.0068	1.0000	-0.0321	0.9536
CO-UNC	-0.0033	1.0000	0.0054	1.0000

Supplementary Table 5: **Post-hoc tests for all significant session × group interaction effects, reflecting group differences in changes of systems recruitment or integration (4 sessions, 2 groups)**. Tests (two-sample t-tests, two-sided) for differences: 'Naive' vs. 'Early', 'Early' vs. 'Middle', 'Middle' vs. 'Late'. Abbreviations: auditory (AU), cerebellum (CER), cingulo-opercular (CO), default mode (DM), dorsal attention (DA), fronto-parietal (FP), memory (MEM), salience (SAL), somatomotor (SOM), subcortical (SUB), uncertain (UNC), ventral attention (VA), and visual (VIS).

**Correlation with behavior: Naive vs. Late (both groups)**			
Systems	r	$p_{uncorr.}$	p_{FDR}
$\Delta d'$ and Δ recruitment			
SAL	0.3377095	0.028723065	1
DM	0.3354091	0.029898227	1
$\Delta d'$ and Δ integration			
FP-SAL	0.3530235	0.021836677	1
MEM-VIS	0.3215687	0.037836231	1
SOM-VA	0.3198655	0.038922327	1
DM-FP	-0.3099325	0.04577489	1
DM-SAL	-0.4142908	0.006378749	0.5740874
AU-MEM	-0.4412674	0.003442311	0.3132503
Δ pRT and Δ recruitment			
DM	-0.3478555	0.02398683	1
VIS	-0.3378605	0.02864729	1
Δ pRT and Δ integration			
AU-MEM	0.3178699	0.04022718	1
CER-DM	0.3405356	0.02733201	1
CO-UNC	-0.3630498	0.01812347	1
CO-VIS	0.3338558	0.03071395	1
DM-FP	0.3560102	0.02066934	1

Supplementary Table 6: **Correlations between the change in network dynamics and the change in behavior**. Pearson correlation coefficient (r) between the across-session changes (Naive vs. Late) in recruitment (or integration) and the across-session changes in d' ($\Delta d'$) and pRT (Δ pRT) observed for both the experimental and control groups. Abbreviations: auditory (AU), cerebellum (CER), cingulo-opercular (CO), default mode (DM), dorsal attention (DA), fronto-parietal (FP), memory (MEM), salience (SAL), somatomotor (SOM), subcortical (SUB), uncertain (UNC), ventral attention (VA), and visual (VIS).

Correlation with behavior: Naive vs. Early (experimental)			
Systems	r	$p_{uncorr.}$	p_{FDR}
$\Delta d'$ and Δ integration			
AU-VA	0.4903851	0.0240127927	1
CER-DA	0.4585068	0.0365743723	1
CO-DA	0.4957494	0.0222872683	1
CO-SUB	-0.515905	0.0166670006	1
DA-SOM	0.710518	0.0003068366	0.02792213
DA-SUB	0.4700412	0.0315444066	1
DM-SAL	0.4558561	0.037814337	1
FP-SOM	0.449137	0.0411045974	1
Δ pRT and Δ integration			
AU-VA	-0.4498745	0.04073297	1
CER-DA	-0.4463136	0.042551842	1
CO-DA	-0.4645735	0.033856106	1
CO-SUB	0.5106186	0.018016262	1
DA-SOM	-0.5641078	0.007729913	0.7034221
UNC-VA	0.5189307	0.015932156	1

Supplementary Table 7: **Relationship between the change in network dynamics and the change in behavior**. Pearson correlation coefficient (r) between the changes in recruitment (or integration) and the changes in d' ($\Delta d'$) and pRT (Δ pRT) during early phase of training (Naive vs. Early) of the experimental group. Abbreviations: auditory (AU), cerebellum (CER), cingulo-opercular (CO), default mode (DM), dorsal attention (DA), fronto-parietal (FP), memory (MEM), salience (SAL), somatomotor (SOM), subcortical (SUB), uncertain (UNC), ventral attention (VA), and visual (VIS).

**Repeated measures MLM: session × goup interaction (GLM)**			
System	Statistics		
	χ^2	$p_{uncorr.}$	p_{FDR}
SAL	19.289622	0.000238	0.003096
VIS	12.21532	0.006681	0.043425
DM	10.220241	0.016784	0.060016
UNC	9.538138	0.022929	0.060016
FP	9.523477	0.023083	0.060016
MEM	8.635576	0.03455	0.074858
VA	7.979876	0.046429	0.086226
DA	5.976738	0.112747	0.183215
SOM	4.77535	0.189006	0.273008
CO	4.233999	0.23728	0.298229
SUB	4.085796	0.252347	0.298229
CER	3.326098	0.344027	0.372696
AU	2.794743	0.424366	0.424366

Supplementary Table 8: **Results of the multilevel modeling (MLM) analysis reflecting session × group interaction effects for systems activity estimated with a standard GLM (2-back vs. 1-back contrast, two-sided)**. In all cases, random intercepts were estimated. The significance of models was estimated with chi-square tests, where models with increasing complexity were compared and the resulting value of Likelihood Ratio Test (χ^2) and corresponding p-value (uncorrected and FDR-corrected) were reported[3]. Abbreviations: auditory (AU), cerebellum (CER), cingulo-opercular (CO), default mode (DM), dorsal attention (DA), fronto-parietal (FP), memory (MEM), salience (SAL), somatomotor (SOM), subcortical (SUB), uncertain (UNC), ventral attention (VA), and visual (VIS).

**Planned contrasts: session × group interaction effect (GLM)**						
System	Naive vs. Early		Naive vs. Middle		Naive vs. Late	
	β	p	β	p	β	p
SAL	-1.09808	0.000812	-1.317647	0.000069	-0.956718	0.003351
VIS	0.121433	0.684265	-0.80268	0.00806	-0.002907	0.992229
DM	-0.750571	0.01187	-0.759561	0.010918	-0.779999	0.009004
UNC	-0.255562	0.27055	-0.689108	0.003441	-0.206578	0.372717
FP	-0.679019	0.095265	-1.235466	0.002735	-0.695647	0.087521
MEM	-0.574095	0.111209	-1.02874	0.004776	-0.401063	0.264535
VA	-0.404449	0.195934	-0.76255	0.01565	-0.732185	0.020184

Supplementary Table 9: **Planned contrasts for all significant session × group interaction effects, reflecting group differences in changes of systems activity estimated with a standard GLM (2-back vs. 1-back contrast, two-sided)**. Contrasts (two-sample t-tests, two-sided): 'Naive' vs. 'Early', 'Naive' vs. 'Middle', 'Naive' vs. 'Late'. Abbreviations: auditory (AU), cerebellum (CER), cingulo-opercular (CO), default mode (DM), dorsal attention (DA), fronto-parietal (FP), memory (MEM), salience (SAL), somatomotor (SOM), subcortical (SUB), uncertain (UNC), ventral attention (VA), and visual (VIS).

3 Supplementary Methods

3.1 Penalized reaction time calculation

To measure behavioral performance in the dual n-back scanning sessions, we incorporated *penalized reaction time* (pRT), which is a measure previously introduced by[4]. This measure combines both measures of accuracy and response time. For every subject, session, task condition, and stimulus modality (auditory, spatial), pRT was defined as:

$$pRT = \frac{1}{n}\sum_{i=1} x_i, \tag{1}$$

where n is the sum of all subject responses and incorrect response omissions, and x_i was obtained from the following formula:

$$x_i = \begin{cases} RT_i, \text{ if answer was correct} \\ 2000, \text{ if answer was incorrect} \\ 2000, \text{ if the correct response was omitted} \end{cases} \tag{2}$$

where RT_i is reaction time of the response during the i-th trial and the scalar value of 2000 is a penalty for an incorrect answer or for the lack of an answer, which is the maximum possible time to respond during each n-back trial measured in milliseconds. For each participant, we calculated average pRT for both modalities to represent a cumulative measure of performance during the dual n-back task.

3.2 Behavioral variability analysis

To assess measures of behavioral variability, we calculated (1) block-wise variants of the two behavioral performance measures, d' and penalized reaction time (pRT), and (2) the standard deviation of these measures over task blocks. For consistency with the measures used in the main text, for both block-wise measures we considered the average value over both stimulus modalities (visual and auditory). This procedure resulted in two measures of block-to-block behavioral variability for each participant and session: the standard deviation of d' ($\sigma_{d'}$) and the standard deviation of pRT (σ_{pRT}). We then used a multilevel analysis to investigate group $\times$ session interactions. Note that these measures of behavioral variability can potentially capture two distinct effects: (1) more or less consistent performance during the 1-back or 2-back blocks, and (2) greater or lesser decreases in behavioral performance from the 1-back to the 2-back condition. Both effects of more consistent performance during a single task condition and a lesser decrease in performance from the 1-back to the 2-back condition would result in an overall decrease in the behavioral variability measures of $\sigma_{d'}$ and σ_{pRT}.

3.3 Anatomical data processing in fMRIPrep

Each T1w (T1-weighted) volume was corrected for INU (intensity non-uniformity) using N4BiasFieldCorrection v2.1.0[5] and skull-stripped using antsBrainExtraction.sh v2.1.0 (employing the OASIS template). Brain surfaces were reconstructed using recon-all from FreeSurfer v6.0.1[6], and the brain mask estimated previously was refined with a custom variation of the method to reconcile ANTs-derived and FreeSurfer-derived segmentations of the cortical gray-matter of Mindboggle[7]. Spatial normalization to the ICBM 152 Nonlinear Asymmetrical template version 2009c[8] was performed through nonlinear registration with the antsRegistration tool of ANTs v2.1.0[9], using brain-extracted versions of both the T1w volume and template. Brain tissue segmentation of cerebrospinal fluid (CSF), white matter (WM), and gray matter was performed on the brain-extracted T1w using FAST[10](FSL v5.0.9).

3.4 Multilevel community detection for signed networks

We ran multilayer community detection on networks with both positive and negative edges[11,12], to investigate whether the antagonism between large-scale systems (reflected by anticorrlated time-series) could influence the recruitment and integration values. First, we defined $N \times N$ matrix A^+_{ijs} by zeroing negative elements of A_{ijs} and $N \times N$ matrix A^-_{ijs} by zeroing positive elements of A_{ijs}. We used this decomposition to represent both A_{ijs} and the corresponding null model p_{ijs} as a linear combination of networks with positive and networks with negative edges:

$$A_{ijs} = A^+_{ijs} - A^-_{ijs} \tag{3}$$

$$\gamma_s p_{ijs} = \gamma^+_s p^+_{ijs} - \gamma^-_s p^-_{ijs} \tag{4}$$

Then, we maximized following modularity quality function:

$$Q_{\pm} = \frac{1}{\mu} \sum_{ijsr} \left[\left(A^{+}_{ijs} - \gamma^{+}_{s} \frac{k^{+}_{is} k^{+}_{js}}{2m^{+}_{s}} \right) - \left(A^{-}_{ijs} - \gamma^{-}_{s} \frac{k^{-}_{is} k^{-}_{js}}{2m^{-}_{s}} \right) \right] \delta_{is} \delta_{ir} \tag{5}$$

With this approach we consider the negative network edges as separate networks when calculating within-layer modularity.

3.5 Standard GLM analysis

To enable reference to the prior literature on the effects of working memory training on activation patterns, we additionally performed a standard General Linear Model (GLM) analysis. In the first level of the GLM analysis, we compared 2-back vs. 1-back activation patterns (two-sided) for all subjects to identify brain areas activated and deactivated in a more difficult 2-back condition. Then, we ran a second-level GLM analysis to investigate consistent patterns of task activation in all sessions and both groups. To make GLM analysis comparable with our functional connectivity analysis, we calculated the mean z-score for the first-level */beta* maps for each ROI from the Power et al.[1] parcellation (Supplementary Figure 10). Then, for all large-scale systems we calculated the mean z-score that reflected the effect size for each network, and sorted them from the lowest to the highest. Next, we used multilevel modelling to test for session $\times$ group interactions for each system (see Supplementary Figure 11 and Supplementary Table 8-9).

References

[1] Power, J. D. *et al.* Functional network organization of the human brain. *Neuron* **72**, 665–678 (2011).

[2] Schaefer, A. *et al.* Local-global parcellation of the human cerebral cortex from intrinsic functional connectivity mri. *Cerebral Cortex* 1–20 (2017).

[3] Field, A., Miles, J. & Field, Z. *Discovering statistics using R* (Sage publications, 2012).

[4] Ginestet, C. E. & Simmons, A. Statistical parametric network analysis of functional connectivity dynamics during a working memory task. *Neuroimage* **55**, 688–704 (2011).

[5] Tustison, N. J. *et al.* N4itk: improved n3 bias correction. *IEEE transactions on medical imaging* **29**, 1310–1320 (2010).

[6] Dale, A. M., Fischl, B. & Sereno, M. I. Cortical surface-based analysis: I. segmentation and surface reconstruction. *Neuroimage* **9**, 179–194 (1999).

[7] Klein, A. *et al.* Mindboggling morphometry of human brains. *PLoS computational biology* **13**, e1005350 (2017).

[8] Fonov, V. S., Evans, A. C., McKinstry, R. C., Almli, C. & Collins, D. Unbiased nonlinear average age-appropriate brain templates from birth to adulthood. *NeuroImage* S102 (2009).

[9] Avants, B. B., Epstein, C. L., Grossman, M. & Gee, J. C. Symmetric diffeomorphic image registration with cross-correlation: evaluating automated labeling of elderly and neurodegenerative brain. *Medical image analysis* **12**, 26–41 (2008).

[10] Zhang, Y., Brady, M. & Smith, S. Segmentation of brain mr images through a hidden markov random field model and the expectation-maximization algorithm. *IEEE transactions on medical imaging* **20**, 45–57 (2001).

[11] Traag, V. A. & Bruggeman, J. Community detection in networks with positive and negative links. *Physical Review E* **80**, 036115 (2009).

[12] Zhang, H., Wang, C.-D., Lai, J.-H. & Philip, S. Y. Modularity in complex multilayer networks with multiple aspects: a static perspective. In *Applied Informatics*, vol. 4, 7 (SpringerOpen, 2017).
