# Supplementary material for: Dynamic reconfiguration of functional brain networks during working memory training
Source: Nat Commun. 2020 May 15;11:2435. doi: 10.1038/s41467-020-15631-z (PMC7229188; doi:10.1038/s41467-020-15631-z)
Supplement: Supplementary file 3 — Reporting Summary [file 41467_2020_15631_MOESM3_ESM.pdf]

nature research

Corresponding author(s): Karolina Finc

Last updated by author(s): Jan 12, 2020

Reporting Summary

Nature Research wishes to improve the reproducibility of the work that we publish. This form provides structure for consistency and transparency in reporting. For further information on Nature Research policies, see Authors & Referees and the Editorial Policy Checklist .

Statistics

For all statistical analyses, confirm that the following items are present in the figure legend, table legend, main text, or Methods section.

n/a	Confirmed	
☐	☒	The exact sample size (*n*) for each experimental group/condition, given as a discrete number and unit of measurement
☐	☒	A statement on whether measurements were taken from distinct samples or whether the same sample was measured repeatedly
☐	☒	The statistical test(s) used AND whether they are one- or two-sided<br>*Only common tests should be described solely by name; describe more complex techniques in the Methods section.*
☐	☒	A description of all covariates tested
☐	☒	A description of any assumptions or corrections, such as tests of normality and adjustment for multiple comparisons
☐	☒	A full description of the statistical parameters including central tendency (e.g. means) or other basic estimates (e.g. regression coefficient) AND variation (e.g. standard deviation) or associated estimates of uncertainty (e.g. confidence intervals)
☐	☒	For null hypothesis testing, the test statistic (e.g. F, t, r) with confidence intervals, effect sizes, degrees of freedom and P value noted<br>*Give P values as exact values whenever suitable.*
☒	☐	For Bayesian analysis, information on the choice of priors and Markov chain Monte Carlo settings
☐	☒	For hierarchical and complex designs, identification of the appropriate level for tests and full reporting of outcomes
☐	☒	Estimates of effect sizes (e.g. Cohen's *d*, Pearson's *r*), indicating how they were calculated

Our web collection on statistics for biologists contains articles on many of the points above.

Software and code

Policy information about availability of computer code

Data collection	Neuroimaging data were collected using a GE Discovery MR750 3 Tesla MRI scanner (General Electric Healthcare) with a standard 8-channel head coil. Structural images were collected using a three-dimensional high resolution T1-weighted gradient-echo (FSPGR BRAVO) sequence (TR = 8.2 s, TE = 3.2 ms, FOV = 256 mm, flip angle = 12 degrees, matrix size 256 x 256, voxel size = 1 x 1 x 1 mm, 206 axial oblique slices). Functional scans were obtained using a T2*-weighted gradient-echo, echo- planar imaging (EPI) sequence sensitive to BOLD contrast (TR = 2,000 ms, TE = 30 ms, FOV = 192 mm, flip angle = 90 degrees, matrix size = 64 x 64, voxel size 3 x 3 x 3 mm, 0.5 mm gap). For each functional run, 42 axial oblique slices were acquired in an interleaved acquisition scheme, and 5 dummy scans (10 s) were obtained to stabilize magnetization at the beginning of the EPI sequence. Behavioral responses were collected using Presentation 17.2. software (Neurobehavioral Systems, Albany, NY).

Data analysis	Functional and anatomical data were structured according to the BIDS (Brain Imaging Data Structure) standard (Gorgolewski et al., 2016), and validated with BIDS Validator (v0.26.9, https://bids-standard.github.io/bids-validator/). Neuroimaging data was preprocessed using fMRIPrep 1.1.1 (Esteban et al., 2019) - a Nipype based tool, AFNIv16.2.07, MCFLIRT(FSL v50.0.9, FreeSurfer v6.0.1, ANTs v2.1.0, https://fmriprep.readthedocs.io/en/latest/workflows.html.<br><br>Non-smoothed functional images were denoised using Nilearn 0.4.2 (Abraham et al., 2014) and Nistats 0.0.1, and custom code (https://osf.io/wf85u/(DOI 10.17605/OSF.IO/WF85U).<br><br>Static modularity was calculated using BCT package in MATLAB R2017a. Multilayer modularity was calculated using genLouvain algorithm (Mucha et al., 2010) in MATLAB R2017a.<br><br>Multilevel model analysis was performed using nlme (Pinheiro et al., 2019) package in R (version 3.4.4) .<br><br>All code available at at: https://github.com/kfinc/wm-training-modularity

For manuscripts utilizing custom algorithms or software that are central to the research but not yet described in published literature, software must be made available to editors/reviewers. We strongly encourage code deposition in a community repository (e.g. GitHub). See the Nature Research guidelines for submitting code & software for further information.

Data

Policy information about availability of data

All manuscripts must include a data availability statement. This statement should provide the following information, where applicable:

- Accession codes, unique identifiers, or web links for publicly available datasets
- A list of figures that have associated raw data
- A description of any restrictions on data availability

The raw behavioral data and fMRI results are available for download at https://osf.io/wf85u/ (DOI 10.17605/OSF.IO/WF85U). The source data underlying Figures 2-7, and Supplementary Figures 1-21 are provided as a Source Data file. The raw fMRI data are available from the corresponding author on request.

Field-specific reporting

Please select the one below that is the best fit for your research. If you are not sure, read the appropriate sections before making your selection.

☒ Life sciences ☐ Behavioural & social sciences ☐ Ecological, evolutionary & environmental sciences

For a reference copy of the document with all sections, see nature.com/documents/nr-reporting-summary-flat.pdf

Life sciences study design

All studies must disclose on these points even when the disclosure is negative.

Sample size	Total N = 53. Seven participants did not complete training procedure, therefore the final sample consisted of 46 healthy volunteers (23 control group; 23 experimental group).
Data exclusions	Seven participants did not complete the study: one due to brain structure abnormalities detected at the first scanning session, and six due to not completing the training procedure. We excluded four high motion participants (2 from the control group, and 2 from the experimental group) with a mean FD larger than 0.2 mm and more than 10 % of outlier volumes in any scanning session.
Replication	We replicated our results using different parcallation, behavioral measures and dynamic analysis based on signed matrices.
Randomization	After the first fMRI scan, participants were randomly assigned to one of the two training groups: experimental and control.
Blinding	The study was double-blind; the experimenter performing the fMRI examination was not aware of the group assignment of the participants, and participants were not aware that the study was designed in a way that there were two groups (experimental and control).

Reporting for specific materials, systems and methods

We require information from authors about some types of materials, experimental systems and methods used in many studies. Here, indicate whether each material, system or method listed is relevant to your study. If you are not sure if a list item applies to your research, read the appropriate section before selecting a response.

Materials & experimental systems

n/a	Involved in the study
x	Antibodies
x	Eukaryotic cell lines
x	Palaeontology
x	Animals and other organisms
	x Human research participants
x	Clinical data

Methods

n/a	Involved in the study
x	ChIP-seq
x	Flow cytometry
	x MRI-based neuroimaging

Human research participants

Policy information about studies involving human research participants

Population characteristics	Fifty-three healthy volunteers (26 female; mean age: 21.17; age range: 18--28 years). All participants were right-handed, had normal or corrected-to-normal vision, and had no hearing deficits. Seven participants did not complete the study: one due to brain structure abnormalities detected at the first scanning session, and six due to not completing the training procedure. The final sample consisted of forty-six participants who completed the entire training procedure, participated in all four fMRI scanning sessions, and had no history of neurological or psychiatric disorders nor gross brain structure abnormalities. Each group consisted of 23 subjects with no group differences in age (two-sample t-test: $t(42.839) = 0.22$, $p = 0.83$) or fluid intelligence (two-sample t-test: $t(42.882) = 0.51$, $p = 0.61$) as measured by Raven's Advanced Progressive Matrices (RAPM).
Recruitment	Participants were recruited from the local community through word-of-mouth and social networks
Ethics oversight	Informed consent was obtained in writing from each participant, and ethical approval for the study was obtained from the Ethics Committee of the Nicolaus Copernicus University Ludwik Rydygier Collegium Medicum in Bydgoszcz, Poland, in accordance with the Declaration of Helsinki.

Note that full information on the approval of the study protocol must also be provided in the manuscript.

Magnetic resonance imaging

Experimental design

Design type	Block-design, resting-state
Design specifications	Four fMRI scanning sessions per subject. Each session of the task consisted of 20 blocks (30 s per block; 12 trials with 25% of targets) of alternating 1- and 2-back conditions.
Behavioral performance measures	Response time, button press. Behavioral performance was calculated using d' and penalized reaction time (pRT) measures. We tested whether any subject exceed +/- SD from the mean value of these measures.

Acquisition

Imaging type(s)	Functional, structural
Field strength	3
Sequence & imaging parameters	T1-weighted gradient-echo, T2*-weighted gradient-echo, echo-planar imaging (EPI)
Area of acquisition	Whole-brain
Diffusion MRI	☐ Used ☒ Not used

Preprocessing

Preprocessing software	fMRIPrep 1.1.1
Normalization	Spatial normalization to standard spaces is performed using ANTs' (antsRegistration) in a multiscale, mutual-information based, nonlinear registration scheme.
Normalization template	MNI
Noise and artifact removal	Non-smoothed functional images were denoised using Nilearn 0.4.2 (Abraham et al., 2014) and Nistats 0.0.1. We implemented voxel-wise confound regression by regressing out (1) signals from six aCompCor components, (2) 24 motion parameters representing 3 translation and 3 rotation timecourses, their temporal derivatives, and quadratic terms of both, (3) outlier frames with FD > 0.5mm and DVARS (Power et al., 2010) with a threshold of +/- 3 SD, together with their temporal derivatives, (4) task effects and their temporal derivatives (Whitfield-Gabrieli & Nieto-Castanon, 2012) and (5) any general linear trend. Time-series were filtered using 0.008-0.25 Hz band-pass filter.

Volume censoring: Outlier frames with FD > 0.5mm and DVARS with a threshold of +/- 3 SD.

Statistical modeling & inference

Model type and settings: We used two-level (trials nested within participants) and three-level (trials nested within sessions nested within participants) multilevel models (MLM). In all cases, random intercepts were estimated. The significance of models was estimated with chi-square tests, where models with increasing complexity were compared and the resulting value of Likelihood Ratio Test and corresponding p-value.

Effect(s) tested: Main effect of group, session, condition, session x group, session x condition x group interaction.

Specify type of analysis: ☐ Whole brain ☐ ROI-based ☒ Both

Anatomical location(s): Power 264 ROIs, Schaefer 300 ROIs parcellation

Statistic type for inference (See Eklund et al. 2016): n/a

Correction: FDR, Bonferroni

Models & analysis

n/a	Involved in the study	
☒	☐	Functional and/or effective connectivity
☐	☒	Graph analysis
☒	☐	Multivariate modeling or predictive analysis

Graph analysis: Normalized modularity and multilayer modularity on weighted graphs.
